# Adiposome Lipidomic Shifts Following Bariatric Surgery in Association With Weight Loss and Cardiometabolic Benefit

**DOI:** 10.1210/clinem/dgaf586

**Published:** 2025-10-24

**Authors:** Elsayed Metwally, Lundon Burton, Imaduddin Mirza, Mohammed H Morsy, Giorgia Scichilone, Amro Mostafa, Yuko Shimotake, Melani Lighter, Francesco M Bianco, Chandra Hassan, Mario A Masrur, Brian T Layden, Abeer M Mahmoud

**Affiliations:** Department of Medicine, Division of Endocrinology, Diabetes, and Metabolism, College of Medicine, University of Illinois Chicago, Chicago, IL 60612, USA; Department of Cytology and Histology, Faculty of Veterinary Medicine, Suez Canal University, Ismailia 41522, Egypt; Department of Medicine, Division of Endocrinology, Diabetes, and Metabolism, College of Medicine, University of Illinois Chicago, Chicago, IL 60612, USA; Department of Medicine, Division of Endocrinology, Diabetes, and Metabolism, College of Medicine, University of Illinois Chicago, Chicago, IL 60612, USA; Department of Medicine, Division of Endocrinology, Diabetes, and Metabolism, College of Medicine, University of Illinois Chicago, Chicago, IL 60612, USA; Department of Medicine, Division of Endocrinology, Diabetes, and Metabolism, College of Medicine, University of Illinois Chicago, Chicago, IL 60612, USA; Department of Pharmacology, College of Medicine, University of Illinois Chicago, Chicago, IL 60612, USA; Department of Surgery, College of Medicine, University of Illinois Chicago, Chicago, IL 60612, USA; Department of Surgery, College of Medicine, University of Illinois Chicago, Chicago, IL 60612, USA; Department of Surgery, College of Medicine, University of Illinois Chicago, Chicago, IL 60612, USA; Department of Surgery, College of Medicine, University of Illinois Chicago, Chicago, IL 60612, USA; Department of Surgery, College of Medicine, University of Illinois Chicago, Chicago, IL 60612, USA; Department of Medicine, Division of Endocrinology, Diabetes, and Metabolism, College of Medicine, University of Illinois Chicago, Chicago, IL 60612, USA; Jesse Brown Veterans Affairs Medical Center, Chicago, IL 60612, USA; Department of Medicine, Division of Endocrinology, Diabetes, and Metabolism, College of Medicine, University of Illinois Chicago, Chicago, IL 60612, USA; Department of Kinesiology and Nutrition, College of Applied Health Sciences, University of Illinois Chicago, Chicago, IL 60607, USA

**Keywords:** adiposomes, extracellular vesicles, lipidomics, bariatric surgery, obesity, cardiometabolic risk

## Abstract

**Context:**

Adiposomes carry bioactive lipids that shape systemic metabolism and vascular function.

**Objective:**

Building on our previous findings that obese adiposomes are ceramide/free fatty acid–enriched and linked to dysfunction, we tested whether bariatric surgery remodels adiposome lipidomes and whether these shifts track with metabolic and vascular improvements.

**Methods:**

Twenty-three obese individuals were assessed before and after bariatric surgery. Clinical evaluations included body mass index (BMI), body composition, glycemic markers, inflammatory markers, and vascular function. Adipose tissue biopsies were collected, and adiposomes were isolated for non-targeted lipidomic analysis using mass spectrometry.

**Results:**

Bariatric surgery induced significant reductions in BMI, visceral fat, HOMA-IR, inflammatory markers, and systolic blood pressure, alongside marked improvements in vascular function. Adiposome lipidomics identified 550 species across 19 classes; 124 increased and 66 decreased after surgery. Class-level shifts showed decreases in triglycerides, diglycerides, cholesteryl esters, phosphatidic acids, fatty acids, and acylcarnitines, with increases in phosphocholines, phosphoethanolamines, lysophosphatidylcholines, sphingomyelins, ultralong-chain ceramides, and fatty acid–hydroxy fatty acids (FAHFAs); these changes were larger in participants with ≥20% BMI reduction. Changes in 36 lipid species correlated with BMI change: triglycerides 18:1/20:4/22:6 positively, and sphingomyelins (SM) d40:1, SM d42:4, and phosphatidylcholine 17:0/18:2 inversely. Improvements in nitric oxide, vascular reactivity, and adiponectin tracked with rises in ultralong-chain ceramide/FAHFAs/phospholipids and declines in acylcarnitine/cholesterol esters/diacylglycerols/phosphatidic acids. Machine learning models predicted BMI from lipid features with >80% accuracy (*R^2^* >0.90; mean absolute error <0.3), highlighting SM d40:1, SM d42:4, and fatty acid 17:1 as top contributors. Pathway enrichment analysis linked these changes to sphingolipid signaling and glycerophospholipid metabolism.

**Conclusion:**

Bariatric surgery remodels adiposome lipids, and these shifts align with improvements in cardiometabolic risk markers.

Obesity is a global health crisis posing severe threats to both individual health and healthcare systems worldwide. It is characterized by the excessive accumulation of adipose tissue and is strongly associated with serious comorbidities, including type 2 diabetes mellitus, cardiovascular disease, and metabolic syndrome (1, 2). Despite extensive efforts to combat obesity through lifestyle interventions such as diet and exercise, sustained weight loss and meaningful metabolic improvements continue to evade many individuals (3, 4). While pharmacological therapies provide additional treatment avenues, persistent long-term efficacy and safety concerns limit their widespread use (5). For individuals with severe obesity, bariatric surgery has emerged as the most effective therapeutic approach, offering sustained weight loss and profound improvements in metabolic health (6, 7). Surgical procedures such as Roux-en-Y gastric bypass and sleeve gastrectomy function as weight loss treatments and powerful metabolic interventions. Beyond caloric restriction alone, bariatric surgery significantly enhances insulin sensitivity, attenuates systemic inflammation, and promotes beneficial lipidomic alterations. These metabolic improvements result from a complex interplay of mechanisms, including altered gut hormone signaling, modified bile acid metabolism, shifts in gut microbiota composition, and substantial reductions in adipose tissue mass (8).

An emerging area in understanding the metabolic benefits of bariatric surgery involves adipose-derived extracellular vesicles (adiposomes), which mediate intercellular communication by transferring bioactive lipids, proteins, and nucleic acids between cells and tissues (9-11). Our prior studies revealed that adiposomes isolated from obese individuals drive endothelial dysfunction by compromising caveolae integrity and impairing nitric oxide (NO) signaling in endothelial cells (12, 13). Moreover, our recent findings identified elevated ceramide levels and decreased phospholipid content in adiposomes from obese individuals compared to lean controls, highlighting the potential role of adiposomes in obesity-related vascular and metabolic impairments (9).

Given their involvement in metabolic regulation, analyzing adiposome lipidomic profiles before and after bariatric surgery offers valuable insights into how weight loss reshapes systemic lipid metabolism and influences cardiometabolic risk. Numerous studies initially focused on proteins and miRNAs, identifying cargo that can modulate inflammatory signaling and insulin pathways. However, recent attention has shifted to the lipid composition, given the pivotal role of bioactive lipids in cell signaling, inflammation, and metabolism (14). Among the lipid species commonly identified in extracellular vesicles are phospholipids, which are essential components of cell membranes and lipoproteins; sphingolipids, which regulate insulin signaling, inflammatory pathways, and cell viability; fatty acid–hydroxy fatty acids (FAHFAs), which exhibit potential anti-inflammatory and insulin-sensitizing properties; and others such as fatty acids, acylcarnitines, and triglycerides (15). Comprehensive characterization of these lipid species via adiposome lipidomics provides novel mechanistic insights into the molecular transformations underlying metabolic improvements induced by bariatric surgery.

This study aimed to comprehensively characterize the lipidomic shifts in adiposomes following bariatric surgery and explore their associations with cardiovascular and metabolic improvements. By leveraging advanced mass spectrometry techniques, we sought to identify specific lipid classes and species that undergo significant remodeling, thus shedding light on potential biomarkers or mechanistic pathways of metabolic improvement. Additionally, integrated analyses involving pathway enrichment and neural network modeling were employed to explore the predictive roles of adiposome lipids for metabolic indices after bariatric surgery.

## Methods

### Subject Enrollment

Twenty-three obese adults (20 female, 3 male) were enrolled. Inclusion criteria were body mass index (BMI) ≥30 kg/m^2^, age between 19 and 50 years, and scheduled sleeve gastrectomy at the University of Illinois Hospital in Chicago. Baseline blood samples and physiological measurements were collected 2 to 3 weeks prior to surgery, and subcutaneous adipose tissue (SAT) samples were obtained intraoperatively. Follow-up assessments, including repeat blood collection, physiological measurements, and adipose tissue sampling, were conducted 3 months after surgery. Exclusion criteria included pregnancy, smoking, prior bariatric surgery, and diagnoses of liver, renal, or heart failure, cancer, or autoimmune disease. Lifestyle and medication questionnaires are collected within our institutional bariatric cohort at each study visit. For this analysis, we excluded individuals who reported tangible lifestyle changes beyond standard postoperative guidance (eg, enrollment in a weight loss program, initiation of structured exercise regimens exceeding routine advice) or major medication alterations between baseline and 3 months. All participants were instructed not to initiate nonstandard interventions during the 3-month postoperative window. The study adhered to the Declaration of Helsinki and was approved by the University of Illinois Institutional Review Board (protocol #2021-1113). Participants provided written informed consent.

### Cardiometabolic Risk Measurements

Anthropometric data (body weight, BMI) were recorded, and body composition (fat vs lean mass) was assessed using dual-energy x-ray absorptiometry (iDXA, General Electric Inc.). Fasting glucose, insulin, and hemoglobin A1c (HbA1c) were measured using established protocols (16). Insulin resistance was estimated via the homeostasis model assessment (HOMA-IR), calculated as fasting insulin (U/L) × fasting glucose (nmol/L)/22.5 (17). Serum lipids, including total cholesterol, low-density lipoprotein (LDL), high-density lipoprotein (HDL), and triglycerides, were measured using enzymatic assays (16, 18, 19) (Roche Diagnostics, Indianapolis, IN, USA). Routine liver function tests (alanine aminotransferase [ALT], aspartate aminotransferase [AST], bilirubin, albumin, alkaline phosphatase) were conducted in the hospital's clinical laboratory. Plasma nitric oxide (NO) levels were quantified using a nitrate/nitrite assay kit (Cayman Chemical, Ann Arbor, MI) (20-22). Circulating inflammatory markers, including interleukin (IL)-6 and CRP, were measured using high-sensitivity immunoassays (R&D Systems, Minneapolis, MN, USA). Serum leptin and adiponectin concentrations were assessed with human ELISA kits (RRIDs: AB_2127617 for IL-6 and AB_3657710 for CRP) (Leptin: KAC228 (AB_2892779); Adiponectin: KHP0041 (AB_3101887); Invitrogen, Waltham, MA, USA). Liver steatosis was evaluated using the Aplio i-series Attenuation Imaging (ATI) package (Aplio i900, Canon Ultrasound Systems, Melville, NY, USA), with a low-frequency curvilinear transducer used to visualize the right hepatic lobe and calculate attenuation coefficients (dB/cm/MHz) within defined regions of interest.

### Sample Acquisition

On the day of bariatric surgery, blood samples were collected before the induction of anesthesia. Following anesthesia, SAT samples were obtained intraoperatively by the surgeon. All samples were immediately placed in cold 2-(4-(2-hydroxyethyl)piperazin-1-yl)ethanesulfonic acid (HEPES) buffer to preserve tissue viability. At the 3-month postoperative visit, participants fasted for 12 hours prior to sample collection. A subcutaneous fat biopsy was then performed by a certified nurse practitioner from the gluteal region under local anesthesia, and the tissue was promptly transferred to cold HEPES buffer for further processing.

### Adiposome Isolation

Adipose tissue samples were processed following protocols previously described by our group (12), with minor modifications. Briefly, tissues were rinsed in sterile Medium 199 (Gibco, Waltham, MA, USA), minced into small fragments, and enzymatically digested using Type I collagenase (Worthington) in Medium 199 containing 4% bovine serum albumin (BSA). The resulting suspension was filtered and centrifuged at 500*g* to separate mature adipocytes, which were then cultured on transwell inserts in Medium 199 supplemented with 5% exosome-depleted fetal bovine serum (FBS) and 1% penicillin/streptomycin. After 24 to 48 hours of incubation, the conditioned medium was sequentially centrifuged (1000*g* for 5 minutes; 15 000*g* for 15 minutes), filtered through a 0.45-μm membrane, and ultracentrifuged at 150 000*g* for 2 hours to isolate adiposomes. Pelleted adiposomes were resuspended and quantified using a Nanoparticle Tracking Analyzer (NanoSight NS300, Malvern Instruments Ltd, UK). Protein extraction from adiposomes was performed using RIPA buffer, and total protein was measured using the Pierce BCA assay (ThermoFisher Scientific). Proteins were separated via 4% to 12% Bis-Tris SDS-PAGE, transferred onto PVDF membranes, and probed overnight with primary antibodies against CD9 (AB_940926), CD81 (AB_943630), PPARγ (AB_777392), adiponectin (AB_1523093), FABP4 (AB_10563156), and apolipoprotein B (AB_2056954) (Abcam, Waltham, MA, USA). Detection was carried out using IRDye-labeled secondary antibodies (anti-mouse [AB_10956588] and anti-rabbit [AB_621843]) and (LI-COR Biosciences) and visualized on an Odyssey Clx infrared imaging system (700 nm for IRDye680 and 800 nm for IRDye800).

### Lipid Extraction and Liquid Chromatography–Mass Spectrometry Lipidomic Analysis

Adiposome lipids were isolated using a modified Folch extraction protocol (12). The lipid extracts were analyzed via liquid chromatography–mass spectrometry (LC-MS) utilizing an Agilent 6545 Q-TOF system equipped with an Agilent Poroshell C18 column (2.1 × 100 mm, 2.7-μm particle size; Agilent Technologies, Santa Clara, CA) at 300 µL/min flow rate. Chromatographic separation began with 70% solvent B (0-1 minute), increased to 86% B (3.5-10 minutes), and reached 100% B (11-17 minutes), followed by re-equilibration for 5 minutes. Mass spectrometry settings included a VCap of 3000 V, fragmentor voltage at 145 V, sheath gas flow at 12 L/min (350 °C), and drying gas at 11 L/min (200 °C). Lipid identification was conducted using Agilent Lipid Annotator software, which built an MS/MS library based on precursor m/z and retention time matching. Molecular feature extraction was performed in Agilent Profinder (vB.10.00), identifying peaks above 5000 counts, quality scores above 60, and at least 2 isotopic signals. Retention time alignment was maintained within ±0.1 minute, mass accuracy ≤5 ppm, and peaks integrated using Agilent integrator. Processed data were baseline-corrected, normalized to internal standards, and transferred to Mass Profiler Professional (v15.1, Agilent) for downstream analysis.

### Vascular Function Assessment

Brachial artery flow-mediated dilation (FMD) was assessed using a high-resolution vascular ultrasound system (Aplio i900; Canon Ultrasound Systems, Melville, NY, USA). A pneumatic cuff was positioned on the participant's forearm and inflated to 220 mmHg for 5 minutes. Arterial diameters were recorded 1 minute before cuff inflation (baseline) and again 5 minutes after cuff release during the reactive hyperemia phase. Image analysis was performed using Automated Edge Detection software. FMD (%) was calculated as: [(peak postdeflation diameter − baseline diameter)/baseline diameter] × 100, following established guidelines (16, 18, 21-23). To evaluate flow-induced dilation (FID) in resistance arterioles, small vessels were isolated from adipose tissue samples, meticulously cleaned of connective tissue, and mounted in a pressure myograph chamber following previously established protocols (18-22, 24). Vessels were secured to glass micropipettes using nylon ties and perfused with oxygenated Krebs buffer while exposed to stepwise intraluminal pressure increments ranging from 10 to 100 cm H_2_O. Vessel diameter changes were continuously monitored under an inverted Olympus microscope. To induce a consistent pre-constricted state, arterioles were treated with endothelin-1 (10⁻⁶ mol/L). FID responses were expressed as percent changes in lumen diameter relative to the constricted baseline diameter across each pressure step.

### Statistical Analysis

All statistical analyses were conducted using SPSS (v26.0; SPSS Inc., Chicago, IL) and RStudio (v4.4.1). Continuous variables are reported as mean ± SD, while categorical variables are presented as counts and percentages. The normality of distribution was evaluated using the Shapiro-Wilk test and confirmed with histograms and Q-Q plots. Depending on distribution characteristics, either parametric (paired *t* test) or nonparametric (Wilcoxon signed-rank test) methods were applied to compare pre- and postsurgical values within the same individuals. The Benjamini-Hochberg false discovery rate (FDR) correction was applied to account for multiple testing. Log2 fold changes (Log2 FC) were calculated by dividing postsurgical lipid expression by presurgical levels, followed by log_2_ transformation. Linear regression models, adjusted for demographic covariates, were used to examine associations between changes in individual lipid species and corresponding changes in clinical measures such as BMI. For all regression analyses, all lipid features were standardized (z-scored) within the analytic cohort by mean-centering and scaling to unit variance (mean = 0, SD = 1). Reported beta coefficients (β), therefore, represent the effect of a 1 SD shift in lipid abundance on the clinical variable of interest. Principal component analysis (PCA) was performed in RStudio on paired, scaled data (mean = 0, SD = 1) using the prcomp function with singular value decomposition (SVD). Hierarchical clustering was conducted using the Ward method on paired, normalized lipidomic profiles to identify patterns of change pre- and postintervention. Clustered heatmaps were generated using the heatmap function to visualize participant-level shifts in lipid profiles over time. Data visualization tools included ggplot2 for bar plots, box plots, and volcano plots; correlation matrices were generated using the corrplot package. For paired continuous variables (pre- vs postsurgery), bivariate relationships were tested using Spearman correlation, accounting for within-subject changes. Treemaps were created using the treemap package to display hierarchical lipid class distributions by change magnitude and significance. Lipids that met the significance threshold (FDR <0.05) from the paired analysis were submitted to Lipid Pathway Enrichment Analysis (LIPEA) for pathway mapping, using Fisher's exact test to determine overrepresentation in KEGG metabolic and signaling pathways.

Artificial neural network (ANN) models were developed using IBM SPSS (version 29) to predict postsurgical cardiometabolic risk factors based on paired adiposome lipidomic profiles. Input variables included the quantified concentrations of individual lipid species isolated from adiposomes, measured pre– and post–bariatric surgery. Response variables encompassed obesity, type 2 diabetes, hypertension, dyslipidemia, liver steatosis, vascular dysfunction, and elevated systemic inflammation. A multilayer perceptron architecture with one hidden layer was used, employing sigmoid activation functions and trained using the scaled conjugate gradient algorithm. Input features were standardized prior to model fitting. The class imbalance was addressed by assigning inverse-proportional class weights. A 70/30 train-test split was implemented with 10-fold cross-validation to reduce overfitting and ensure model generalizability. Model performance was evaluated based on accuracy, coefficient of determination (R^2^), and mean absolute error (MAE). For each model, the top 10 most informative lipid predictors were extracted based on variable importance scores, allowing the identification of key lipid species that contributed most significantly to the classification or regression outputs. Feature selection and outcome-specific model parameters were optimized to ensure robust and interpretable predictions of the clinical variables under investigation.

## Results

### Clinical and Anthropometric Changes Following Bariatric Surgery

This study evaluated 23 participants undergoing bariatric surgery for clinical and metabolic parameters before surgery and 3 months postoperatively. A comprehensive summary of these findings is presented in Table 1. The results demonstrated significant weight reduction, reflected by a decrease in average BMI from 46.1 kg/m^2^ to 37.1 kg/m^2^. Substantial reductions were also observed in total body fat percentage and visceral fat mass, accompanied by notable decreases in waist circumference, indicative of reduced central adiposity. Moreover, key metabolic indices improved significantly following surgery: fasting plasma glucose levels declined from prediabetic and diabetic ranges to near-normal values, fasting insulin concentrations substantially decreased, and insulin resistance, assessed by homeostasis model assessment (HOMA-IR), improved markedly (from ∼4.8 to ∼2.0). HbA1c levels improved (from ∼6.2% to ∼5.4%), underscoring enhanced glycemic control.

**Table 1.** Clinical characteristics in obese participants before vs after bariatric surgery

Group	Presurgery (n = 23)	Postsurgery (n = 23)	*P*
Age, years	37 ± 7.7		
BMI, kg/m^2^	46.1 ± 6.1	37.1 ± 5.7	<.001
Body fat, %	51.5 ± 6.6	41.1 ± 6.4	<.001
Visceral fat mass, kg	2.2 ± 0.6	1.3 ± 0.4	<.001
Waist circumference, cm	131.7 ± 6.8	114.5 ± 9.1	<.001
Cardiometabolic function indices			
Fasting plasma glucose, mg/dL	108.7 ± 29.4	75.7 ± 8.5	.004
Fasting plasma insulin, µU/mL	17.0 ± 7.3	10.8 ± 2.1	<.001
HOMA-IR, mIU/L	4.8 ± 3.0	2.0 ± 0.4	<.001
HbA1c, %	6.2 ± 1.0	5.4 ± 0.2	<.001
Total cholesterol, mg/dL	166.4 ± 37.8	151.5 ± 30.7	.200
LDL, mg/dL	92.9 ± 28.3	79.4 ± 14.4	.113
HDL, mg/dL	44.6 ± 11.7	47.2 ± 3.7	.019
Triglycerides, mg/dL	137.0 ± 58.1	119.9 ± 33.3	.058
Plasma bilirubin, mg/dL	0.45 ± 0.21	0.54 ± 0.13	.042
Alkaline phosphatase, U/L	77.4 ± 15.6	67.0 ± 9.4	.003
Plasma ALT, U/L	23.4 ± 26.5	16.2 ± 5.1	.094
Plasma AST, U/L	19.9 ± 16.0	15.3 ± 4.2	.083
Total albumin, g/dL	3.9 ± 0.6	4.2 ± 0.4	.014
Total hemoglobin, g/dL	12.2 ± 1.2	12.8 ± 1.6	.043
Heart rate, BPM	82 ± 15	75 ± 10	.020
Systolic BP, mmHg	135 ± 18	121 ± 9	.002
Diastolic BP, mmHg	79 ± 11	76 ± 6	.192
Nitric oxide, μmol/L	3.4 ± 1.6	4.5 ± 0.9	.004
IL-6, pg/mL	23.0 ± 9.8	11.6 ± 4.6	<.001
Serum CRP, mg/dL	4.0 ± 1.4	2.6 ± 1.1	<.001
Serum leptin, ng/mL	33.1 ± 17.5	12.2 ± 7.2	<.001
Serum adiponectin, μg/mL	5.4 ± 2.6	9.8 ± 6.0	<.001
Leptin: adiponectin ratio	7.2 ± 4.7	1.7 ± 1.3	<.001
Cardiometabolic risk factors			
Abdominal obesity, *n* (%)	23 (100)	23 (100)	1.000
Liver steatosis, *n* (%)	13 (56.5)	10 (43.5)	.237
High ALT, *n* (%)	6 (26.1)	1 (4.3)	.035
Dyslipidemia, *n* (%)	15 (65.2)	6 (26.1)	.008
Hyperglycemia, *n* (%)	10 (43.5)	3 (13.0)	.022
Insulin resistance, *n* (%)	19 (82.6)	1 (4.3)	<.001
High BP, *n* (%)	10 (43.5)	4 (17.4)	.051

Abbreviations: ALT, alanine transaminase; AST, aspartate transaminase; BMI, body mass index; BP, blood pressure; BPM, beat per minute; CRP, C-reactive protein HbA1c, hemoglobin A1C; HDL, high-density lipoprotein; HOMA-IR, homeostasis model assessment of insulin resistance; IL-6, interleukin 6; LDL, low-density lipoprotein; n, number.

Lipid profiles showed favorable changes following bariatric surgery, including increased HDL levels and a downward trend in triglycerides. Although reductions in total cholesterol and LDL were modest, dyslipidemia prevalence markedly decreased from 65.2% to 26.1%, indicating clinically meaningful improvements in lipid abnormalities. Additionally, systemic inflammation significantly decreased after surgery, reflected by lower IL-6 and CRP levels, signifying reduced chronic inflammation characteristic of obesity. Markers of liver function showed favorable trends, with significant decreases in alkaline phosphatase and slight but clinically acceptable elevations in bilirubin, accompanied by nonsignificant reductions in ALT and AST.

**Figure 1.**
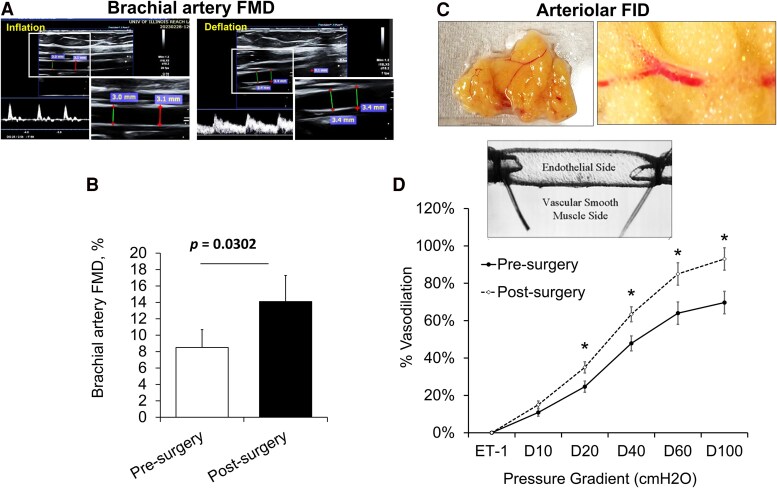
Evaluation of vascular function before and after bariatric surgery. (A) Representative ultrasound images of the brachial artery at baseline (Inflation) and during peak reactive hyperemia (Deflation) following cuff release, used to assess flow-mediated dilation (FMD). Insets display magnified measurements of arterial diameter during inflation and post-hyperemia. (B) Percent change in brachial artery FMD before and after surgery (n = 23. (C) Representative image of freshly excised human adipose tissue used for resistance arteriole isolation. (D) Percent change in flow-induced dilation (FID) of isolated adipose arterioles in response to increasing intraluminal pressure (10-100 cmH_2_O), pre- and postsurgery (n = 23). Data are presented as mean ± standard error (SE); *P* < .05.

Adipokine profiles improved substantially, with leptin levels decreasing dramatically from an average of 33.1 ng/mL to 12.2 ng/mL, while adiponectin levels nearly doubled. As a result, the leptin-to-adiponectin ratio declined markedly from 7.2 to 1.7, indicating a shift toward a more favorable metabolic state. Additionally, participants exhibited significant improvements in vascular function. Brachial artery FMD increased by 66%, indicating enhanced endothelial responsiveness (Fig. 1A and 1B). Resistance arterioles isolated from visceral adipose tissue also demonstrated improved flow-induced dilation (FID), with a 33% increase observed at Δ 60 cmH_2_O pressure gradient, reflective of physiological flow conditions (Fig. 1C and 1D). These vascular enhancements were accompanied by elevated circulating nitric oxide levels, supporting the restoration of endothelial signaling and vascular health. Additionally, systolic blood pressure declined significantly (from 135 to 121 mmHg), while diastolic pressure remained unchanged. Heart rate also decreased, reflecting improved cardiovascular efficiency.

### Global Shifts in Adiposome Lipidomic Profiles Following Bariatric Surgery

**Figure 2.**
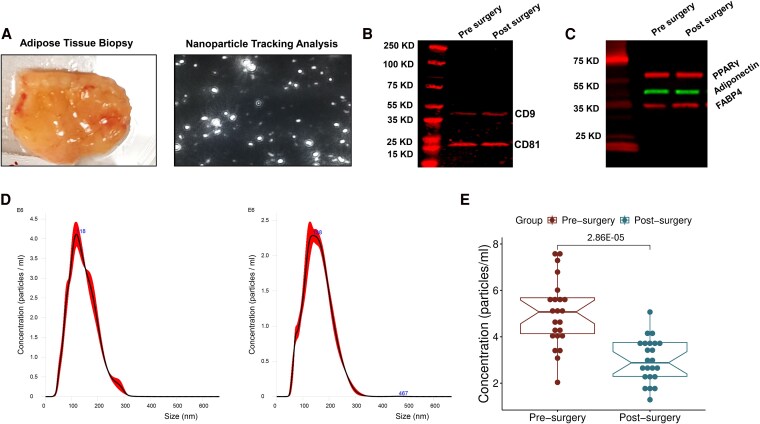
Characterization of human adiposomes before and after bariatric surgery. (A) Representative images showing subcutaneous adipose tissue (SAT) biopsy samples and nanoparticle tracking analysis (NTA) of isolated adiposomes. (B and C) Representative Western blot analysis demonstrating the presence of extracellular vesicle (EV) markers (CD9 and CD81) and adipocyte-specific proteins (PPARγ, adiponectin, FABP4) in isolated adiposomes. (D) Representative NTA distribution curve of adiposome particles before and after bariatric surgery. (E) A box-and-whisker plot comparing adiposome concentration before and after surgery (n = 23).

To examine adiposome lipid changes after bariatric surgery and their association with cardiometabolic improvements, human adiposomes were isolated from SAT (Fig. 2A) and characterized using nanoparticle tracking analysis (NTA). Protein characterization confirmed the presence of standard extracellular vesicle markers, including tetraspanins CD9, CD81, and CD63, and showed no detectable contamination from lipoproteins (apolipoprotein B; APOB) (Fig. 2B). Adipocyte-specific markers, PPARγ, adiponectin, and fatty acid–binding protein 4 (FABP4), were also expressed in the isolated adiposomes, validating their adipocyte origin (Fig. 2C). Adiposome sizes ranged from 50 to 350 nm, with significantly reduced concentrations observed postsurgery compared to presurgery (6.3 × 10^11^ particles/mL vs 8.1 × 10^11^ particles/mL, *P* < .001) (Fig. 2D and 2E).

**Figure 3.**
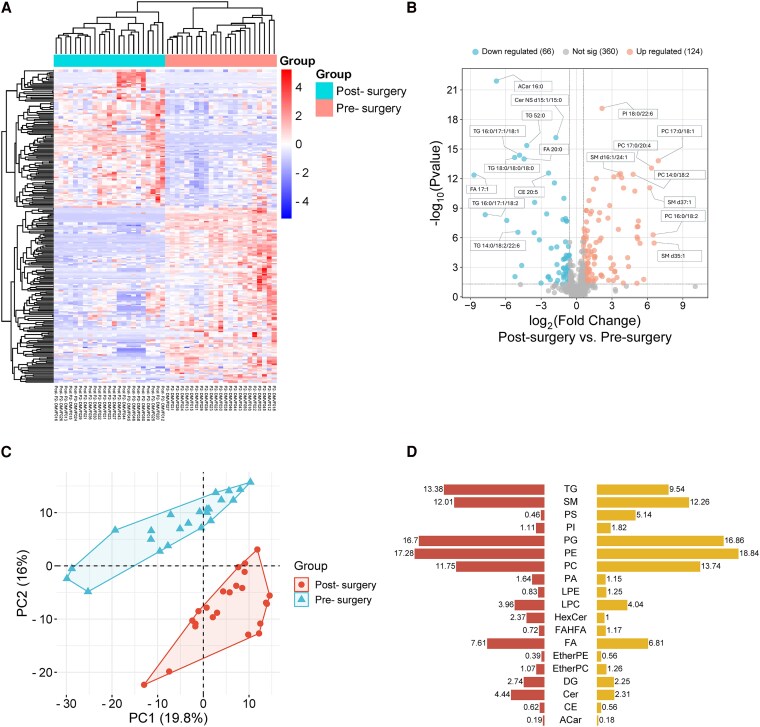
Lipidomic profiling reveals significant alterations in lipid species following surgery. (A) Hierarchical clustering heatmap of lipid species abundances in pre- and postsurgical samples. Each column represents an individual sample, and each row corresponds to a lipid species. Z-score normalized values are shown, with red indicating upregulation and blue indicating downregulation. (B) Volcano plot displaying differential lipid species between postsurgery and presurgery groups. The x-axis shows the log_2_ fold change, and the y-axis shows the −log₁₀(*P*-value). Lipids significantly upregulated (orange, right) or downregulated (blue, left) after surgery are indicated, with selected lipid species annotated. (C) Principal component analysis (PCA) showing distinct clustering of presurgical (upper left) and postsurgical (lower right) samples. The first 2 principal components (PC1 and PC2) account for 19.8% and 16% of the variance, respectively. Ellipses represent 95% CIs for each group. (D) Bar plot summarizing lipid class changes after surgery. Values adjacent to bars indicate the number of significantly altered species per class.

Lipidomic profiling of adiposomes revealed distinct lipid composition differences between pre- and postsurgical states. Hierarchical clustering clearly separated pre- and postsurgical samples, indicating substantial lipid remodeling following surgery (Fig. 3A). The volcano plot further highlighted significant upregulation of multiple lipid species after surgery, alongside notable downregulation of others, reflecting the metabolic adaptations associated with weight loss (Fig. 3B). In total, 550 lipid species across 19 lipid classes were identified. Among these, 124 species significantly increased, and 66 species significantly decreased after surgery (FDR <0.05), adjusted for age, sex, and race/ethnicity. Notably, phosphatidylinositol (PI) 18:0/22:6 significantly increased, while acylcarnitine (ACar) 16:0 significantly decreased postsurgery. Principal component analysis (PCA) further confirmed a clear distinction between pre- and postsurgical lipidomic profiles (Fig. 3C).

Comparison of lipid class composition showed pronounced postsurgical reductions in triglycerides (TG), fatty acids (FA), acylcarnitines (ACar), ceramides (Cer), cholesterol esters (Ce), and diacylglycerols (DG). Conversely, lipids significantly enriched postsurgery included phosphatidylcholine (PC), phosphatidylethanolamine (PE), lysophosphatidylcholine (LPC), sphingomyelins (SM), and fatty acid esters of hydroxy fatty acids (FAHFAs) (Fig. 3D).

### Adiposome Lipidomic Alterations Are Significantly Associated With Postbariatric BMI Reduction

**Figure 4.**
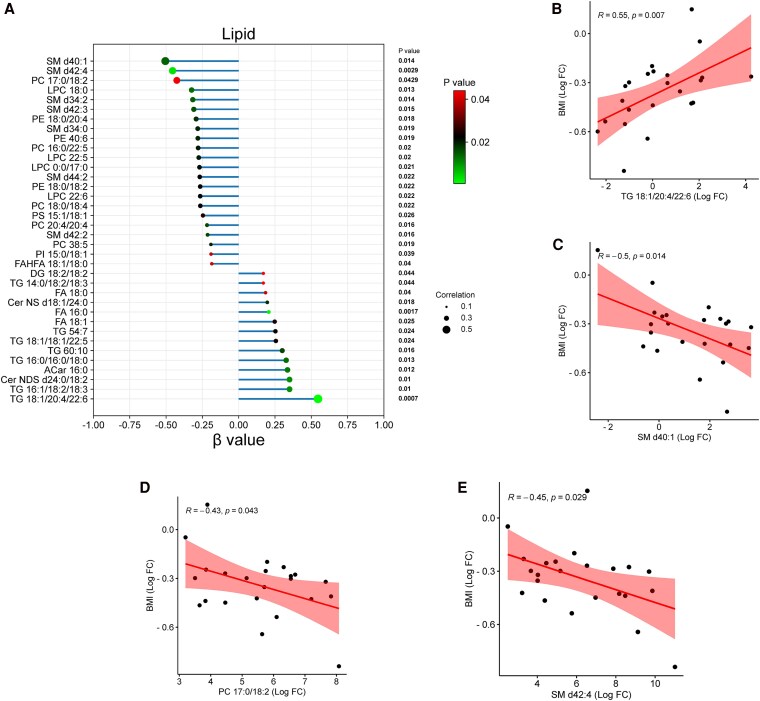
Adiposome lipidomic alterations are significantly associated with postbariatric BMI reduction. (A) Linear regression analysis depicting the relationship between changes in individual lipid species and BMI post–bariatric surgery. The x-axis represents the β values from the regression model (reflecting the slope from univariable models of Δlipid on ΔBMI (within-subject changes), while the y-axis lists the lipid species. Colored dots indicate *P* values, and dot size reflects correlation strength. (B-E) Representative scatter plots showing significant correlations between the log fold-change (Log FC) in the abundance of specific lipid species and the Log FC in BMI. Each point represents an individual subject, with red shaded areas indicating 95% CI. TG 18:1/20:4/22:6 positively correlated with BMI, while SM d40:1, PC 17:0/18:2, and SM d42:4 demonstrated an inverse correlation with BMI.

We performed linear regression analysis to evaluate whether specific adiposomal lipid species were associated with BMI reduction following bariatric surgery (Fig. 4A). The analysis identified 36 lipid species whose postsurgical changes were significantly correlated with changes in BMI. Notably, reductions in certain triglycerides showed strong positive associations with BMI reduction, reflected by positive β values, indicating that greater TG decreases paralleled greater BMI loss. In contrast, several phospholipids and sphingomyelins were inversely correlated with BMI change. For example, TG 18:1/20:4/22:6 levels remained elevated in individuals with less BMI reduction, while SM d40:1 and PC 17:0/18:2 were negatively associated with BMI, suggesting increased levels in those with more substantial weight loss (Fig. 4B-4E). These results support the potential utility of adiposome lipidomic profiles as predictive biomarkers of postsurgical weight loss outcomes.

### Distinct Adiposome Lipid Signatures Following Bariatric Surgery

Comprehensive adiposome lipidomics identified significant differences between pre– and post–bariatric surgery samples. Phospholipid classes PC and PE were increased, with several species showing >5-fold changes (Fig. S1A (25)). As examples, PC 18:2/20:4, LPC 19:0, and PE 18:0/18:2 were significantly increased (Fig. S1B-S1D (25)), with PE 18:0/18:2 showing the most significant change. In contrast, PA species were consistently lower after surgery (Fig. S1E-S1F (25)). Ceramides showed mixed changes: shorter/saturated species (eg, Cer NDS d18:1/24:1) decreased postsurgery, whereas longer-chain (≥39 carbons, eg, Cer NDS d39:1) and unsaturated species (eg, Cer NDS d24:0/18:2) increased (Fig. S2A-S2C (25)). Sphingomyelins were more uniform, with most species increasing (Fig. S2D-S2F (25)). Cholesteryl esters showed species-specific shifts, including decreases in Ce 20:5 and increases in Ce 22:4 (Fig. S2G-I (25)). All TG and DG species showed significant downregulation postsurgery (Fig. S3A-S2E (25)). Fatty acid ester of hydroxy fatty acid (FAHFA) species, such as FAHFA 16:0/22:3, increased postsurgery (Fig. S3F-S3G (25)). Among free fatty acids (FAs), FA 17:1 decreased substantially, whereas those with more unsaturated acyl chains, such as FA 18:2 and FA 18:3, exhibited slight increases (Fig. S3H-S3I (25)).

### Lipidomic Shifts Are Linked to BMI Reduction and Cardiometabolic Risk Factors

**Figure 5.**
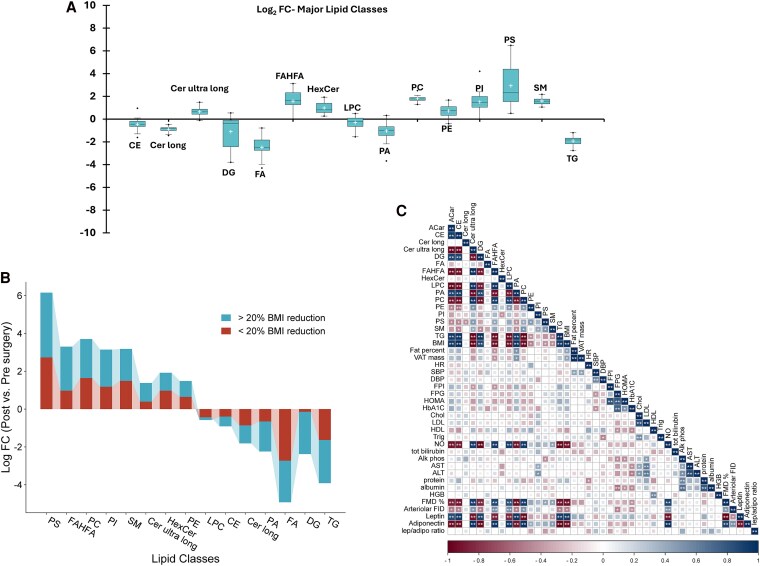
Postsurgical changes in adiposome lipid classes are associated with BMI reduction and cardiometabolic improvements. (A) Box plots showing log fold changes (post- vs presurgery) in total lipid class concentrations across all participants. (B) Stratification of lipid class changes by weight loss response, comparing participants with ≥20% BMI reduction (blue) vs <20% BMI reduction (red). (C) Heat map showing Spearman correlations between changes in total lipid classes and key anthropometric, metabolic, and vascular parameters.

Lipids were summed within each class to assess changes in lipid profiles following bariatric surgery and their associations with BMI reduction and cardiometabolic measures. Totals decreased for Ce, long-chain Cer, TG, DG, PA, and FAs, and increased for ultralong-chain Cer, phospholipids, SM, and FAHFAs (Fig. 5A). These shifts were greater in participants with ≥20% BMI reduction than in those with <20% (Fig. 5B).

Figure 5C presents a heat map of correlations between changes in total lipid classes and anthropometric/cardiometabolic parameters. BMI reductions were positively associated with decreases in ACar, Ce, DG, and PA and inversely with increases in ultralong-chain Cer, FAHFAs, and phospholipids; leptin followed a similar pattern. In contrast, vascular function markers, nitric oxide (NO), brachial FMD, and arteriolar FID, as well as adiponectin, showed the opposite trends, correlating positively with ultralong-chain Cer/FAHFAs/phospholipids and inversely with ACar/Ce/DG/PA. Reductions in fasting plasma insulin were also associated with increases in ultralong-chain Cer and FAHFAs. Overall, this correlation matrix highlights how postsurgical improvements in BMI, metabolic control, and cardiometabolic health parameters can be tightly linked to the magnitude of shifts in adiposome lipid species, providing insight into potential mechanisms and biomarkers of surgical weight loss.

### ANN-based Modeling Links Adiposome Lipids to Obesity and Metabolic Dysfunction

**Figure 6.**
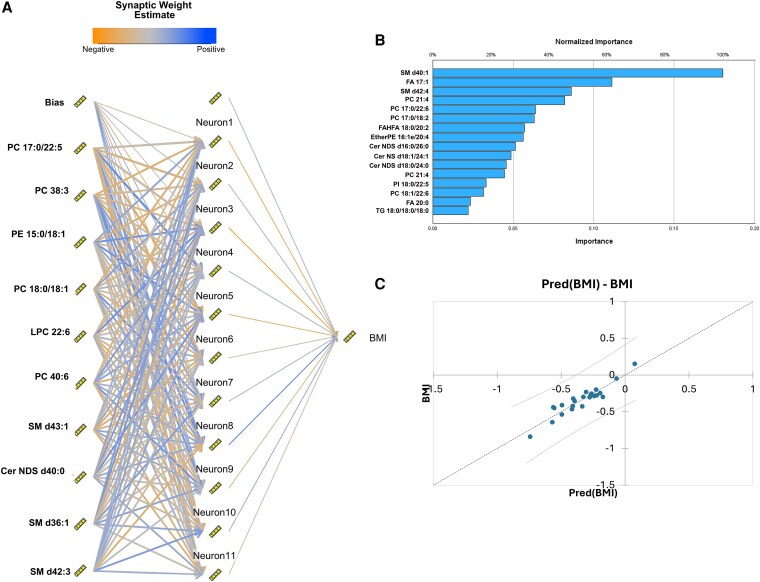
Artificial neural network (ANN) model predicting BMI from adiposomal lipidomic features. (A) Schematic diagram of the ANN model constructed using lipidomic variables as input features. The diagram illustrates the input layer (individual lipid species), a single hidden layer with 11 neurons, and an output layer predicting BMI. (B) Feature importance plot: normalized importance scores of lipid species in predicting BMI, based on the ANN model. (C) Regression plot of predicted vs actual BMI values across test samples. The regression line with 95% confidence intervals demonstrates that the ANN model reliably captures the underlying lipidomic patterns associated with BMI.

An artificial neural network (ANN) was developed to predict BMI changes from lipidomic features (Fig. 6A), and feature importance identified SM d40:1, SM d42:4, and FA 17:1 as top contributors (Fig. 6B). Predicted and observed BMI were strongly correlated, indicating the model effectively captured lipidome-BMI relationships (Fig. 6C). Table S1 (25) summarizes performance across BMI, VAT mass, blood pressure, glucose/insulin indices, and cardiometabolic biomarkers; most models achieved >80% accuracy with R^2^ > 0.90 and low MAE (<0.2-0.3), underscoring the ANN's sensitivity to subtle metabolic variation.

While the top predictive features varied by outcome, SM and Cer consistently emerged across models, alongside PC, FA, and select TG. This recurring pattern suggests these lipid classes are central to predicting both obesity-related traits (eg, BMI, VAT mass) and metabolic dysfunction (eg, insulin resistance, dyslipidemia). Indices of adiposity (BMI, VAT mass) frequently highlight SM and Cer species alongside phospholipids, suggesting links to sphingolipid and phospholipid pathways. Cardiovascular parameters (systolic and diastolic blood pressure, brachial FMD%, and arteriolar FID) were more closely tied to PA, TG, and specific SM/Cer subtypes. Insulin/glucose measures (fasting plasma insulin, fasting plasma glucose, HOMA, HbA1c) were repeatedly associated with unsaturated FAs, PCs, and SMs, consistent with established roles of lipid signaling in insulin resistance. Lipoproteins (Chol, LDL, HDL, Trig) and inflammatory markers (IL-6, CRP) showed variable top predictors across models, most notably including DG, SM, and specific Cer species. These findings highlight the complex interplay between lipid metabolism and systemic inflammation.

### Adiposome Lipidomic Enrichment Analysis Reveals Key Metabolic and Signaling Pathways Underlying Postsurgical Adaptations

**Figure 7.**
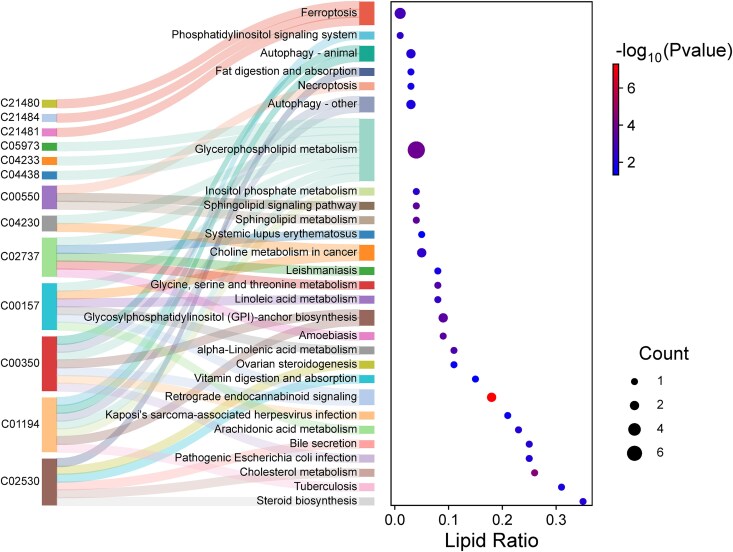
Pathway enrichment analysis of adiposome lipids using LIPEA. The Sankey diagram (left) links individual lipids to enriched KEGG pathways such as glycerophospholipid metabolism, sphingolipid signaling, autophagy, ferroptosis, and necroptosis. The dot plot (right) shows lipid ratio (x-axis), lipid count (dot size), and statistical significance (color gradient, −log₁₀ *P*-value).

To uncover the biological relevance of the lipidomic changes observed after bariatric surgery, pathway enrichment analysis was performed using Lipid Pathway Enrichment Analysis (LIPEA), as illustrated in Fig. 7. This analysis revealed significant associations between adiposome lipid profiles and several central metabolic pathways, including glycerophospholipid metabolism, sphingolipid metabolism, cholesterol metabolism, bile secretion, and essential fatty acid metabolism (linoleic and alpha-linolenic acid pathways). Notably, beyond classical lipid metabolism, multiple signaling and regulatory pathways were enriched, such as phosphatidylinositol signaling, autophagy, necroptosis, and ferroptosis, highlighting the involvement of membrane dynamics and cell fate regulation in postsurgical metabolic adaptation. Immune and inflammation-related pathways were also significantly enriched, including those linked to systemic lupus erythematosus and Kaposi's sarcoma-associated herpesvirus infection. In addition, hormonal and micronutrient-associated pathways, such as vitamin digestion and absorption, ovarian steroidogenesis, and choline metabolism in cancer, were prominently represented. The accompanying dot plot emphasizes the statistical significance (*P* < .01) and lipid contribution ratios of these pathways, with glycerophospholipid and phosphatidylinositol signaling pathways ranking among the most prominent. Collectively, these findings indicate that bariatric surgery triggers a broad, coordinated shift in lipidomic signatures across multiple biological domains, supporting the role of adiposome lipid remodeling in mediating the systemic cardiometabolic benefits of weight loss.

## Discussion

This study provides the first in-depth lipidomic profiling of adiposomes following bariatric surgery, revealing a striking metabolic reprogramming of these vesicles that closely parallels improvements in vascular and metabolic health. We demonstrate that bariatric surgery elicits a robust shift in adiposome lipid composition, characterized by significant depletion of lipotoxic lipids such as short-chain ceramides and diacylglycerols, and a concurrent enrichment of bioactive species including phospholipids, sphingomyelins, and FAHFAs. These lipid changes strongly correlate with enhanced insulin sensitivity, suppressed systemic inflammation, and restored endothelial function. Our findings suggest that adiposome lipidomics may serve as a valuable dual-purpose tool, both as a biomarker of metabolic recovery and as a potential contributor to the cardiometabolic benefits observed after bariatric surgery. These results advance understanding of the molecular changes accompanying surgical weight loss, while highlighting the need for future studies to confirm mechanistic roles and evaluate therapeutic potential in obesity and related comorbidities.

Within 3 months of bariatric surgery, participants experienced striking improvements across multiple cardiometabolic domains. The substantial reduction in BMI, visceral adiposity, and fat mass reflects not only effective weight loss but also a profound shift in central obesity, hallmarks of cardiometabolic risk (7, 26-28). Improvements in glycemic markers, including fasting glucose, insulin, HOMA-IR, and HbA1c, were rapid and significant, supporting earlier studies showing that bariatric surgery can reverse prediabetes and type 2 diabetes through both weight-dependent and independent mechanisms, including enhanced gut hormone and insulin signaling (29-31). Lipid remodeling was also evident, with notable increases in HDL and reductions in triglycerides, consistent with previous findings on the cardiovascular benefits of sleeve gastrectomy (8, 29, 32, 33). Inflammatory burden decreased significantly, as reflected by reduced IL-6 and CRP, reinforcing the notion that bariatric surgery reverses obesity-associated low-grade inflammation and restores vascular health (1, 26, 34, 35). Hepatic parameters trended favorably, suggesting amelioration of subclinical liver steatosis, echoing prior evidence on liver recovery postsurgery (36).

Hormonal shifts, particularly the marked drop in leptin and a 2-fold increase in adiponectin highlight a transition to a more insulin-sensitive, anti-inflammatory profile. The sharp decline in the leptin-to-adiponectin ratio, an emerging marker of metabolic health, adds to the clinical relevance (37-39). Finally, vascular reactivity improved significantly, as evidenced by enhanced brachial FMD and arteriolar FID, accompanied by reductions in blood pressure and resting heart rate and enhanced nitric oxide bioavailability, supporting earlier work linking surgical weight loss to endothelial recovery and autonomic balance (12, 40). These clinical improvements, spanning glycemic control, lipid metabolism, inflammation, hepatic function, and vascular health, showed strong correlations with the observed remodeling of adiposome lipid profiles, underscoring their potential as both biomarkers and mediators of postsurgical metabolic recovery.

The observed reduction in the postbariatric adiposome particle concentration suggests suppression of vesicle biogenesis, potentially reflecting decreased metabolic stress and adipose tissue inflammation after bariatric surgery (41). A key finding of this study is the pronounced shift in adiposome lipid composition following bariatric surgery. Hierarchical clustering and PCA of lipidomic profiles revealed a clear pre- vs postoperative separation, reflecting a systemic lipid shift consistent with previously reported metabolic reprogramming after bariatric surgery (42). Notably, the marked decrease in ceramides, particularly short-chain species such as Cer NDS d18:1/24:1, aligns with their established role in insulin resistance through Akt inhibition and mitochondrial dysfunction (43). Importantly, this reduction paralleled improvements in HOMA-IR, reinforcing the reported contribution of ceramides to metabolic dysfunction (14, 44). These findings are consistent with our previous work demonstrating that ceramide levels in adiposomes from morbidly obese individuals correlated significantly with insulin resistance and vascular dysfunction (9), further supporting their role as mechanistic mediators and potential biomarkers of cardiometabolic disease.

Conversely, the observed increase in long-chain ceramides (eg, Cer NDS d39:1) and sphingomyelins (SMs) may indicate a compensatory shift toward membrane stabilization and cardioprotective signaling (45). Of particular relevance, an increased sphingomyelin-to-ceramide (SM/Cer) ratio has been associated with improved cardiovascular risk factors, including reduced infarct size and better post-ischemic recovery, likely through attenuation of pro-apoptotic ceramide signaling (46, 47). Therefore, the elevated SM/Cer ratio seen in postbariatric adiposomes may not only reflect favorable structural lipid remodeling but also serve as a promising biomarker of enhanced cardiometabolic health. This interpretation is consistent with prior studies, such as Heneghan et al (48), which reported reductions in circulating ceramides and improved insulin sensitivity and inflammation following bariatric surgery. However, our findings uniquely extend these observations to adiposome-specific lipid remodeling, highlighting extracellular vesicles as potential markers and mediators of metabolic recovery that warrant further investigation beyond our observational study.

The enriched phospholipids (PC, PE, LPC) and FAHFAs further support a metabolic shift toward insulin sensitization and inflammation resolution. For example, PC 18:0/22:6, which increased 5-fold postsurgery, has been shown to enhance IRS-1 phosphorylation in hepatocytes (49, 50). FAHFAs, such as FAHFA 18:0/22:3, are potent ligands for PPARγ and GPR120, key regulators of anti-inflammatory signaling and glucose uptake (51, 52). These findings are in line with preclinical studies showing that FAHFAs reverse diet-induced insulin resistance in rodents (53). Our correlation analysis revealed that decreases in BMI and leptin were positively associated with reductions in acylcarnitines, cholesteryl esters, diglycerides, and phosphatidic acid, while negatively associated with increases in ultralong-chain ceramides, FAHFAs, and phospholipids. Improvements in fasting plasma insulin also correlated with these beneficial lipid species, reinforcing the role of lipidomic shifts in metabolic recovery (54). These findings align with prior studies demonstrating that bariatric surgery improves sphingolipid and phospholipid metabolism (55). Such changes are linked to improved adipokine signaling, as reflected by elevated adiponectin and reduced leptin.

Extending beyond metabolic benefits, these lipidomic changes were also closely associated with improvements in vascular function, reflected by enhanced nitric oxide bioavailability, brachial FMD, and arteriolar FID. Increased sphingomyelins, phospholipids, and FAHFAs in adiposomes appear to contribute not only to metabolic regulation but also to endothelial recovery. These findings underscore the coordinated nature of lipid-driven restoration across both vascular and metabolic systems following bariatric surgery. These observations are further supported by our prior study (12), which demonstrated that adiposomes from obese diabetic individuals impair endothelial nitric oxide signaling and disrupt caveolar architecture. These detrimental effects were primarily mediated by the elevated glycated ceramide content within the adiposomes and were significantly diminished when these ceramides were selectively depleted. The current findings suggest that bariatric surgery may reverse such adverse vascular signaling by reprogramming adiposome lipid composition, particularly by reducing ceramides, thereby promoting the emergence of a vasoprotective vesicle phenotype that facilitates endothelial recovery. This proposed mechanism is the focus of our ongoing mechanistic studies.

Earlier studies demonstrate that weight loss re-engages critical metabolic control pathways, such as autophagy, ferroptosis, sphingolipid signaling, and glycerophospholipid metabolism, whose disruption contributes to obesity-related complications (56, 57). In line with these findings, our pathway enrichment analysis revealed that the same pathways are significantly remodeled following bariatric surgery, suggesting that changes in adiposome lipids may reflect the broader systemic reprogramming induced by surgical weight loss. As such, adiposome lipidomics may provide a sensitive readout of the cellular stress responses and metabolic adaptations that characterize postsurgical recovery.

To further explore the predictive potential of these lipidomic shifts, we applied machine learning techniques to identify key lipid species associated with metabolic improvement. The strong performance of the ANN model highlights the promise of lipidomic profiling as a tool for precision medicine in obesity and metabolic diseases. Identification of lipid species such as SM d40:1, FA 17:1, and SM d42:4 as key predictors suggests potential targets for future mechanistic studies and therapeutic intervention. From a systems biology perspective, our integration of machine learning modeling, regression, and pathway analyses identified a consistent set of lipid predictors across cardiometabolic domains, suggesting that specific lipid species (eg, SM d40:1, FA 17:1, PC 17:0/22:6) may serve as sensitive indicators of metabolic state. These findings align with previous systems-level studies identifying extracellular vesicle lipids as central nodes in the dysregulated molecular networks associated with obesity (10, 58).

Several lipid species that were significantly altered in postbariatric adiposomes, most notably ceramides, FAHFAs, and phospholipids, are well-characterized for their roles in modulating metabolic and vascular function. Ceramides, in particular, are known to disrupt insulin signaling by inhibiting Akt activation, impairing GLUT4 translocation, and contributing to insulin resistance, especially under obese conditions (59, 60). Their pro-inflammatory effects extend to the vascular endothelium, where ceramide-laden extracellular vesicles activate NF-κB and NLRP3 inflammasomes, fostering vascular inflammation and oxidative stress (61). Chronic ceramide exposure can also disrupt vascular tone by shifting FMD from nitric oxide (NO)- to ROS-dependent mechanisms (62, 63). In contrast, FAHFAs enhance insulin sensitivity by promoting glucose uptake and suppressing hepatic glucose production (64) while attenuating macrophage-driven inflammation through GPR40 signaling (65). Though direct effects on vascular tone are limited, their anti-inflammatory action may stabilize endothelial function and reduce cytokine-induced vascular leakage (66, 67). Phospholipids, including PC and PE, support membrane integrity and modulate key signaling cascades, such as the PI3K/Akt pathway, enhancing insulin responsiveness and endothelial nitric oxide production (68-70). Moreover, oxidized phospholipids like OxPAPC have been shown to suppress NF-κB activation and cytokine release in endothelial cells (71, 72). Together, these bioactive lipids likely contribute to insulin sensitivity, inflammation, and vascular function, underscoring the potential mechanistic role of adiposome lipid remodeling in metabolic recovery.

Beyond their utility as potential biomarkers, these adiposome lipid changes suggest avenues for therapeutic exploration, subject to mechanistic and interventional confirmation. Given the established bioactivity of lipids such as ceramides, FAHFAs, and phospholipids, manipulating adiposome composition through dietary, pharmacologic, or surgical means may represent a novel strategy to restore metabolic balance in obesity. For example, approaches aimed at selectively depleting pathogenic ceramide species or enriching vesicles with anti-inflammatory lipids like FAHFAs could potentially replicate some of the benefits of bariatric surgery without requiring invasive intervention. Additionally, engineering synthetic vesicles that mimic the beneficial lipid profiles of postsurgical adiposomes may provide a foundation for next-generation, cell-free therapies in cardiometabolic disease.

The observed changes in adiposome lipid content also raise important questions regarding the tissue origin, target specificity, and mechanistic role of these vesicles in inter-organ communication. While adipose tissue is a major contributor to circulating extracellular vesicles, the distinct contributions of subcutaneous vs visceral depots and their lipidomic signatures remain to be fully delineated. Understanding how these remodeled adiposomes interact with recipient cells such as hepatocytes, endothelial cells, or immune populations will be essential to uncover their functional significance. Future studies incorporating extracellular vesicle tracking, lipid flux analysis, and cell-specific functional assays will be critical for establishing causality and clarifying the mechanisms by which adiposome remodeling influences systemic physiology.

While prior research has established that bariatric surgery improves metabolic health through hormonal, microbial, and inflammatory pathways, this study adds a new dimension by showing that extracellular vesicle lipidomics may serve both as a biomarker of recovery and as a potential contributor to the underlying mechanisms, pending confirmation in interventional studies. The complexity of postsurgical adaptation underscores the importance of integrating adiposome with other -omic platforms for a more comprehensive understanding of the molecular networks driving sustained metabolic improvements, or relapses, following bariatric surgery. Moreover, future research should prioritize larger, multicenter cohorts with diverse patient populations and extended longitudinal sampling to assess the consistency and clinical relevance of adiposome lipidomic signatures. Pairing these molecular changes with functional assays and tissue-specific outcomes will be essential to unlock the full translational potential of adiposome profiling in obesity-related diseases.

Despite the strengths of this study, including comprehensive adiposome lipidomic profiling, integration with clinical measurements, and the application of machine learning, several limitations should be acknowledged. First, the sample size, while sufficient for detecting robust lipidomic changes, limits the generalizability of our findings. Larger, multicenter studies with more diverse populations are needed to validate the lipidomic signatures identified here and determine their applicability across different demographic and clinical contexts. Second, the study cohort was predominantly female, with only 2 male participants among the 23 subjects. This sex imbalance may limit the interpretation of sex-specific lipidomic responses and their relevance to male patients. Future studies should ensure balanced sex representation to evaluate potential sex-dependent differences in adiposome lipid composition and metabolic indices. Third, while the study identified correlations between specific lipid species and metabolic or vascular improvements, the causal role of these lipids remains to be fully established. Functional validation of candidate lipids, particularly ceramides and FAHFAs, through in vitro and in vivo models will be necessary to determine their mechanistic impact on target cells such as endothelial cells or smooth muscle cells.

The sampling site was a limitation in this study. Intraoperative tissue was collected from peri-umbilical lower-abdominal SAT, whereas follow-up tissue was from upper-gluteal/lower-back SAT to balance operative feasibility and outpatient safety. Although site-related variation is possible, both are trunk SAT depots; prior reports and our in-house lipidomics show high concordance across these sites, with differences far smaller than trunk–thigh/extremities contrast (73, 74). Consistent with this, within-subject and site-adjusted/site-stratified sensitivity analyses yielded unchanged effect directions and magnitudes, indicating minimal bias from sampling location. Another limitation is the relatively small sample size (n = 23) used for constructing the artificial neural network (ANN) model. Although 10-fold cross-validation was applied to mitigate overfitting, the limited cohort size and potential similarity between folds may have led to overly optimistic performance estimates. Thus, the ANN results should be considered exploratory and require validation in larger, independent cohorts.

Our focus on lipidomics, though informative, provides only a partial view of the complex molecular adaptations following bariatric surgery. Multi-omic integration, including genomic and proteomic analyses, will be essential to build a more complete mechanistic framework and better understand long-term trajectories of metabolic health and disease remission. Finally, we acknowledge that the observed effects reflect the combined impact of bariatric surgery and its standardized postoperative care rather than surgery in isolation. At our institution, postoperative care follows a uniform pathway, diet progression from liquids to pureed/soft and then to a high-protein, reduced-simple-carbohydrate plan, explicitly designed to accommodate reduced gastric volume after surgery. This standard-of-care diet is intrinsic to the surgical intervention (not an extraneous exposure) and was consistently implemented across participants; nevertheless, residual or unmeasured confounding cannot be fully excluded in a self-controlled before-and-after design.

## Conclusion

While causality is not established given the observational design, this study provides descriptive evidence of adiposome lipid remodeling that tracks with systemic postsurgical improvements. Through in-depth profiling, we demonstrate that bariatric surgery induces a distinct remodeling of the adiposome lipidome, marked by reductions in lipotoxic species such as DG and TGs, and enrichment of bioactive lipids, including sphingomyelins, phospholipids, and FAHFAs. Given that elevated DG and TG levels are linked to ectopic fat accumulation and insulin resistance, their postsurgical decline likely contributes to enhanced glycemic control and reduced metabolic stress. Importantly, these lipidomic shifts align with improvements in insulin sensitivity, inflammation resolution, and vascular function, suggesting that adiposome cargo may serve as both a biomarker of metabolic state and a mechanistic mediator of inter-tissue communication during recovery. In particular, changes in sphingolipid and phospholipid content, known regulators of membrane integrity, signaling pathways, and inflammatory tone, emerge as key indicators of metabolic restoration. These findings highlight the adiposome lipidome as a promising platform for precision diagnostics and therapeutic discovery in obesity-related diseases. Overall, our study demonstrates postbariatric remodeling of adiposome lipids and their associations with cardiometabolic risk markers. As the field advances, integrating adiposome lipidomic data with multi-omic and clinical phenotyping may help identify biomarkers and generate hypotheses for preventive and therapeutic strategies.

## Data Availability

The datasets used and/or analyzed during the current study are available from the corresponding author upon reasonable request.

## Abbreviations

ACar: acylcarnitine
ALT: alanine aminotransferase
ANN: artificial neural network
AST: aspartate aminotransferase
BMI: body mass index
CE: cholesterol esters
Cer: ceramides
CRP: C-reactive protein
DG: diacylglycerols
FA: fatty acid
FABP4: fatty acid–binding protein 4
FAHFA: fatty acid ester of hydroxy fatty acid
FDR: false discovery rate
FID: flow-induced dilation
FMD: flow-mediated dilation
HbA1c: glycated hemoglobin
HDL: high-density lipoprotein cholesterol
HOMA-IR: homeostatic model assessment for insulin resistance
IL-6: interleukin 6
LDL: low-density lipoprotein cholesterol
LPC: lysophosphatidylcholine
PC: phosphatidylcholine
PE: phosphatidylethanolamine
PCA: principal component analysis
SAT: subcutaneous adipose tissue
SM: sphingomyelins
TG: triglycerides
VAT: visceral adipose tissue
